# Bioprospecting Indoor Bacteria Producing Bioactive Metabolites

**DOI:** 10.1111/1758-2229.70402

**Published:** 2026-08-23

**Authors:** Maria A. Andersson, Szabolcs Nagy, Balázs Kakasi, Raimo Mikkola, Heidi Salonen

**Affiliations:** ^1^ Department of Civil Engineering Aalto University Espoo Finland; ^2^ Department of Precision Livestock Farming and Animal Biotechnics Institute of Animal Sciences, Hungarian University of Agriculture and Life Sciences Keszthely Hungary; ^3^ Research Institute of Biomolecular and Chemical Engineering, Faculty of Engineering, University of Pannonia Veszprém Hungary; ^4^ International Laboratory for Air Quality and Health, Faculty of Science, School of Earth & Atmospheric Sciences, Queensland University of Technology Brisbane Australia

## Abstract

New biomolecules are prospected for new antimicrobial, antiparasitic and cytostatic drugs. Here, we present bioassay‐guided methods for prospecting indoor bacteria producing non‐protein antibiotic molecules. Out of 650 bacterial colonies tested, 108 antimicrobial isolates representing 15 species and cryptic *Actinobacteria*, produced non‐protein antibiotics affecting mammalian cells. Methanol extracts of the 108 strains tested with a battery of bioassays revealed four toxicity endpoints: (1) Cell death (CD). (2) Boar sperm motility inhibition (BSMI). (3) Inhibition of cell proliferation (ICP). (4) Mitochondrial toxicity (MT). The battery of endpoints divided the 108 strains into four toxicity profiles: Profile I: Lethal toxins (42 strains). Positive controls: Alamethicin, a channel forming ionophore and a biosurfactant surfactin. Profile II: Sublethal mitochondrial toxins (51 strains). Positive controls: The mitochondrial toxins valinomycin and antimycin A. Profile III: Sublethal cytostatic toxins (7 strains). Positive control: Chaetoglobosin A, a glucose transport inhibitor. Profile IV: Cytostatic toxins (8 strains). Positive controls: Actinomycin D, RNA syntesis inhibitor. Strains of *Bacillus, Streptomyces and Paenibacillus* produced amylosin, cereulide, surfactins, lichenysins, iturins, fengysin, valinomycin and fusarisidins. Strains of *Peribacillus Lysinibacillus*, *Microbacterium, Nocardiopsis* and cryptic *Actnobacteria* produced possible novel bioactive molecules. The indoor environments were easily accessible habitats for bacteria producing ionophores, surfactants, and mitochondrial toxins.

## Graphical abstract

The indoor environments served as easily accessible habitats for bacteria producing mitochondrial toxins and ionophores, effects exhibited by known antiparasitic drugs and effects useful in mitochondrial research. This study describes the bacterial species and genera producing toxic peptide antibiotics and identifies some of their biological targets in eukaryotic cells.

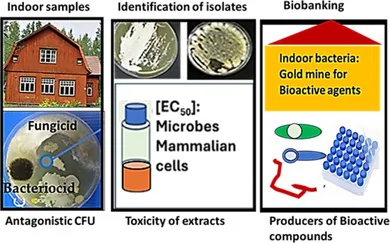

## 1 Introduction

The composition of bacterial species in buildings is related to the use of space, the materials used and the ventilation strategy (Bosch et al. 2024; Gilbert and Stephens 2018; Verdier et al. 2014). These can theoretically be divided into two categories: (1) Metabolically active bacterial communities colonizing moist materials, contributing to the microbial deterioration of building structures (Andersen et al. 2011, 2021; Chudzik et al. 2024). (2) Background bacteriomes consisting of casually traveling airborne bacteria deposited in indoor air and dust (Andersen et al. 2021; Tanaka and Maruyama 2024; Verdier et al. 2014). In indoor dust, which is sheltered from air currents and UV radiation, survival forms of drought‐resistant bacteria may become enriched (Bale et al. 1993; Brągoszewska and Pastuszka 2018; Potts 1994).

Bacteria communicate and adapt to changing environmental conditions by producing bioactive secondary metabolites (Krespach et al. 2023). Secondary metabolites regulate sporulation, spore germination, motility and biofilm formation (Fang et al. 2020; Julkowska et al. 2005; Krespach et al. 2023; Nandy et al. 2007). These molecules may function at low concentrations as signals enhancing the fitness of the producer strain (Aminov 2013; Ekman et al. 2012), but at higher concentrations they may act as antimicrobial, antiparasitic and cytostatic agents and may also be toxic to mammalian cells (Fajardo and Martínez 2008; Romero et al. 2011; Campbell 2012; Jiang et al. 2012; Jamal and Ahmad 2022; Maud et al. 2024; Pimentel‐Elardo et al. 2010; Wolstenholme et al. 2004). Indoor bacteria producing heat‐stable nonprotein toxins have been less extensively studied than indoor mycotoxin‐producing fungi (Lindemann et al. 2022). The hazardous effects of bacterial secondary metabolites on indoor air quality and human health are controversial, but their biological activities arouse interest.

Novel molecules are constantly bio‐prospected for new environmentally friendly biosurfactants, as well as for new antimicrobials and antiparasitic and cytostatic drugs that can help overcome resistance to existing drugs (Kaplan 2004). In addition, bacterial mitochondrial toxins are useful tools for research on mitochondria‐host cell interactions, mitophagy and apoptosis (Chen et al. 2023; Jiang et al. 2012). Microbes producing novel molecules are commonly searched for in extreme and/or natural environments (Afni 2025; Bennur et al. 2014; Jagannathan et al. 2021; Mondol et al. 2013; Srinivasan et al. 2021). However, new bacterial species were also found indoors (Kämpfer et al. 1999, 2000; Vuorio et al. 1999), and some of them have produced new bioactive molecules (Peltola, Andersson, Haahtela, et al. 2001).

The aims of this study were: (a) to evaluate the indoor environment for bioprospecting bacteria producing heat‐stable non‐protein metabolites, (b) to identify such indoor bacteria, and (c) to identify the toxic targets of selected bacterial metabolites in exposed mammalian cells. This article presents indoor environments as a sink for diverse indoor and outdoor bacteria producing bioactive non‐protein metabolites. Parts of the results are based on previously published observations on bioactive metabolites and toxins produced by indoor bacterial isolates. However, this study also presents novel species producing previously unreported toxins and focuses on the screening and characterization methods used for their detection.

## 2 Materials and Methods

### 2.1 Experimental Design

**FIGURE 1:**
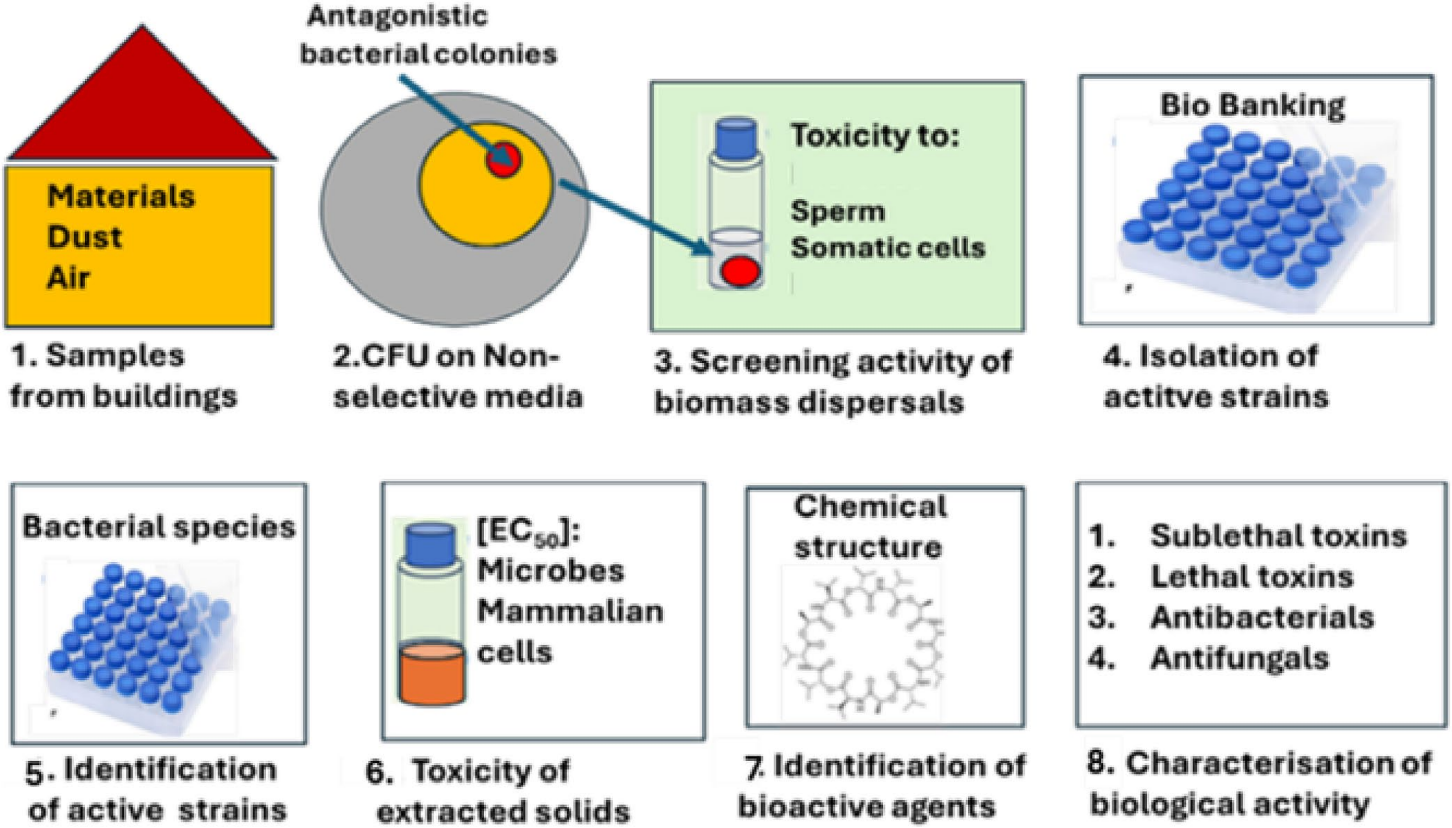
Experimental procedure for screening indoor bacterial colonies producing bioactive molecules, identification of strains producing bioactive metabolites and characterization of their biological activity.

Figure 1 illustrates: (1) Collection of indoor samples. (2) Cultivation of indoor bacteria, targeting antagonistic colonies. (3) Screening biomass dispersals of antagonistic colonies for toxicity against mammalian cells. (4) Biobanking bioactive isolates. (5) Identification of bioactive bacterial isolates. (6) Extracting biomass of bacterial strains, determination of EC_50_ concentrations of extracted substance in bioassays with eukaryotic and microbial cells. (7) Chemical identification of bioactive molecules. (8) Characterizing biological activity of pure substances.

### 2.2 Sample Collection

Twenty‐three samples were collected from a wide range of indoor environments, including day care centres, schools, offices, hospitals and cow sheds in eight Finnish settlements. All sampling sites were associated with complaints related to animal or human health and the indoor environment. Sample collection included air samples (fall out plates, six stage Andersen sampler), dusts (settled on surfaces at 1.5–2 m above the floor level), and construction materials inside buildings and spoiled bedding materials in animal sheds (Table S1. The 23 samples collected from indoor environments of 19 buildings in Finland in the Supporting Information).

### 2.3 Cultivation of Indoor Bacteria and Screening for Antagonistic Colonies According to the “Reverse Alexander Fleming Technique”‐RAFT

**FIGURE 2:**
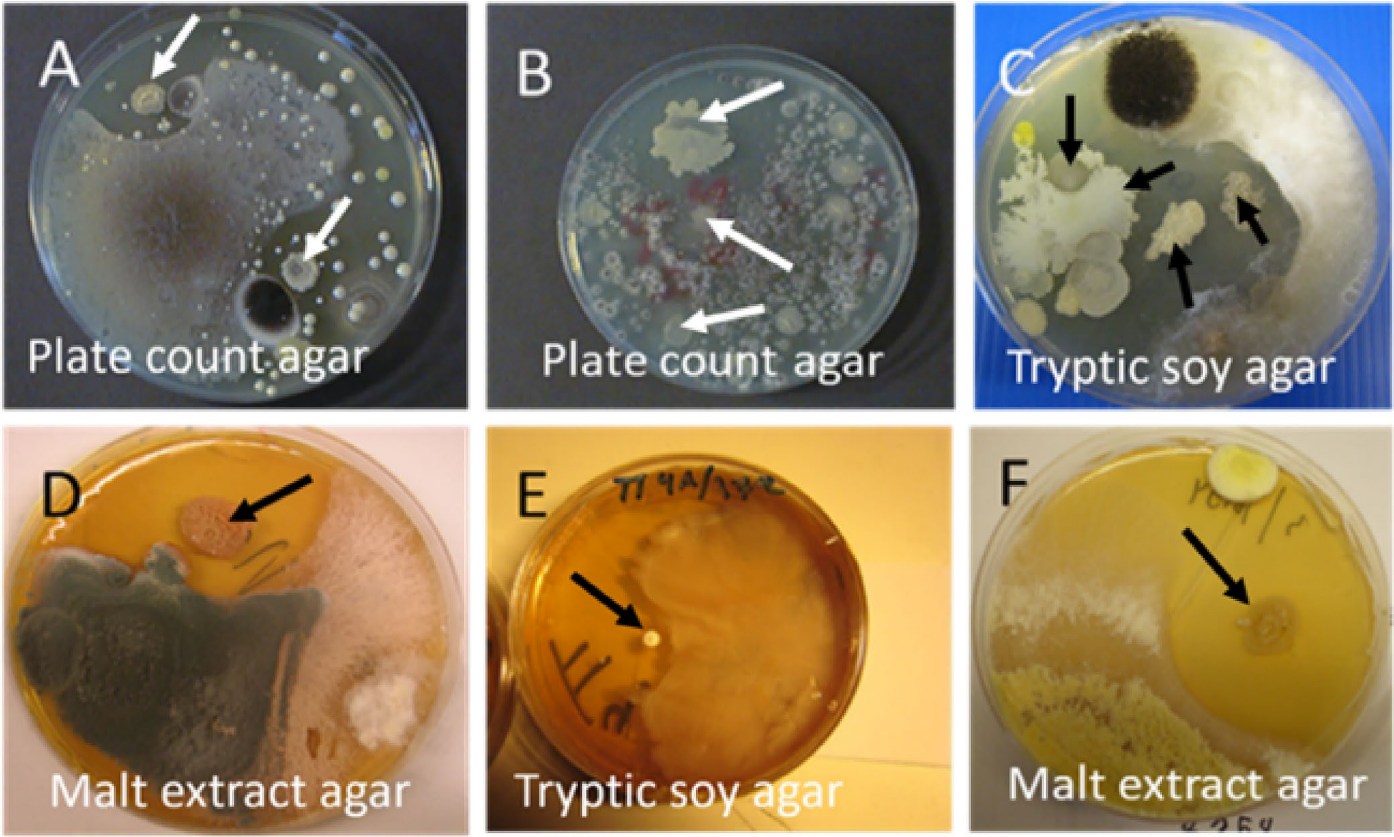
The “reverse Alexander Fleming” technique—RAFT, for isolating bacterial colonies forming inhibition zones against fungal and/or bacterial colonies on the same isolation plate. The plates were sealed with gas permeable tape and incubated for 2–3 weeks at 16°C and at 22°C. Panels A–C show bacterial colonies antagonizing fungi and bacteria. Panel D–F show bacterial colonies antagonistic to fungi.

To increase thebiodiversity of bacterial colonies, samples were cultivated in parallel with and without resuscitation (Andersson et al. 1995) on culture medium with and without reducing sugars: Tryptic soy agar (TSA), and glucose‐containing malt extract agar (MA), from Sharlab Barcelona, Spain, and glucose‐containing plate count agar (PCA), from Becton Dickinson, Sparks, USA as shown in Table 1. TSA was used as a general non‐selective medium, whereas MA and PCA were included to broaden the recovery of physiologically diverse bacteria and thereby increase colony diversity during screening. After incubation for 2–3 weeks at 16°C and 22°C–24°C, inspection focused on colonies forming inhibition zones against nearby fungal or bacterial colonies on the same isolation plate, as shown in Figure 2, Panels A–F. We refer to this approach as the “Reverse Alexander Fleming Technique—(RAFT)” in analogy to Alexander Fleming's isolation of antagonistic *Penicillium* colonies producing antibacterial penicillin compounds. The growth inhibitory effects were examined visually, and biomass of antagonistic colonies was screened for toxicity against mammalian cells (Section 2.4).

**TABLE 1:** Toxicity profiles of commercial toxins that were used as model toxins and as positive controls.

Toxicity profile	Toxin	Toxicity endpoints^a^ EC_50_ μg mL^−1^				References
		BSMI	CD	ICP	MT	
I	Alamethicin	+ (0.6)	+ (0.6)	+ (8)	− None	Salo et al. (2020)
I	Surfactin	+ (5)	+ (5)	+ (20)	− None	Madslien et al. (2013)
II	Valinomycin	+ (< 0.003)	− (> 1)	− (> 1)	+ (0.005)	Salo et al. (2020)
II	Antimycin A	+ (0.005)	− (> 1)	− (> 1)	+ (0.006)	This study
III	Chaetoglobosin A	+ (1)	− (30)	+ (3)	− None	Salo et al. (2020)
IV	Sterigmatocystin	− (> 20)	− (> 20)	+ (0.3)	− None	Salo et al. (2020)
IV	Actinomycin D	− (> 20)	− (> 100)	+ (0.2)	− None	This study
	Triclosan^b^	1	20–50	8	5	Hitikka et al. (2024)

^a^ All tests were run in triplicate and the average differences between triplicate samples were within one dilution step (two‐fold) tested with the same batch of cells maximal difference was one dilution step (SD ≤ 20%).

^b^ Triclosan was used as a general positive control for assay performance and was not used as a model toxin for toxicity profile classification.

### 2.4 Toxicity Testing

#### 2.4.1 Rapid Screening Tests of Biomass of Antagonistic Bacterial Colonies

Toxicity of biomass dispersions from selected colonies was screened using the BSMI (Boar sperm motility inhibiting assay) and ICP (Inhibition of cell proliferation) assays as described in Hitikka et al. (2024). Briefly, 10–20 mg of the colony biomass was suspended in 0.2 mL of methanol and heated for 10 min at 60°C–80°C. A response was scored as positive at 2% biomass dispersion in the BSMI assay and at 4% biomass dispersion in the ICP assay. The commercial extended boar semen was obtained from Figen Ltd. (Tuomikylä, Finland). The continuous cell lines used in the ICP assay, FFL (fetus feline lung cells) and PK‐15 (porcine kidney cells), were provided by the Finnish Food Authority (Helsinki, Finland). Biomass dispersals of the cereulide‐producing *Bacillus cereus* strain NC7041 and the non‐toxic *B. mycoides* strain ATCC 6462 were used as positive and negative controls, respectively. All tests were performed in triplicate, and the average difference of the mean of three measurements performed with the same batch of cells was ≤ 20%, within one dilution step of two‐fold dilutions.

### 2.5 Identification of Isolated Toxic Colonies

Antagonistic colonies screened toxic against mammalian cells were pure‐cultured and identified by whole‐cell fatty acid methyl ester (FAME) analysis using the Sherlock Microbial Identification System with the aerobic TSBA library 3.90 (MIDI Inc., Newark, DE, USA) as described by Pirttijärvi et al. (1998). Identification of selected strains representing each genus and phenotype and toxicity profile was confirmed by ribotyping and physiological characterization, analysis of 16S ribosomal DNA sequences according to Andersson et al. (1998) and Apetroaie et al. (2005).

### 2.6 Determination of the EC_50_ Concentrations of Dry Solids Extracted From Bacterial Biomass

Plate‐grown bacterial biomass (100–300 mg), grown on TSA at 22°C for 1–2 days, was extracted overnight with three volumes of methanol. Extract toxicity was tested in two‐fold dilutions and the toxicity endpoints were recorded as EC_50_ concentrations of methanol‐soluble dry solids. EC_50_ indicates the lowest exposure concentrations that produced the indicated cell damage in > 50% of exposed cells compared to vehicle controls, a value between EC_0_ and EC_100_. Microscopic calculations were confirmed by a 50% change in metabolic readout (medium pH indicating acidosis or alkalosis, resazurin reduction indicating cellular NADH activity, and glucose consumption indicating inhibition or acceleration of glycolysis) compared with vehicle‐only exposure at the same time point. Triclosan (1–50 μg mL^−1^) and methanol (1–2% v/v) were used as positive and negative (vehicle exposure) controls, respectively. Triclosan was used as positive control in all assays. All tests were run in triplicate, the maximal difference of the mean of three measurements was one dilution step of two‐fold dilutions, 20%.

### 2.7 Toxicity Endpoints of Bacterial Extracts in Four Bioassays

EC_50_ concentrations of the dry substances extracted from bacterial biomass were tested in a battery of four bioassays: Ex vivo assays using boar sperm and in vitro assays with somatic primary continuous cell lines. as described below. The assays are described below and their protocols are summarized in Hitikka et al. (2024).

#### 2.7.1 BSMI: Boar Sperm Motility Inhibition Assay

**FIGURE 3:**
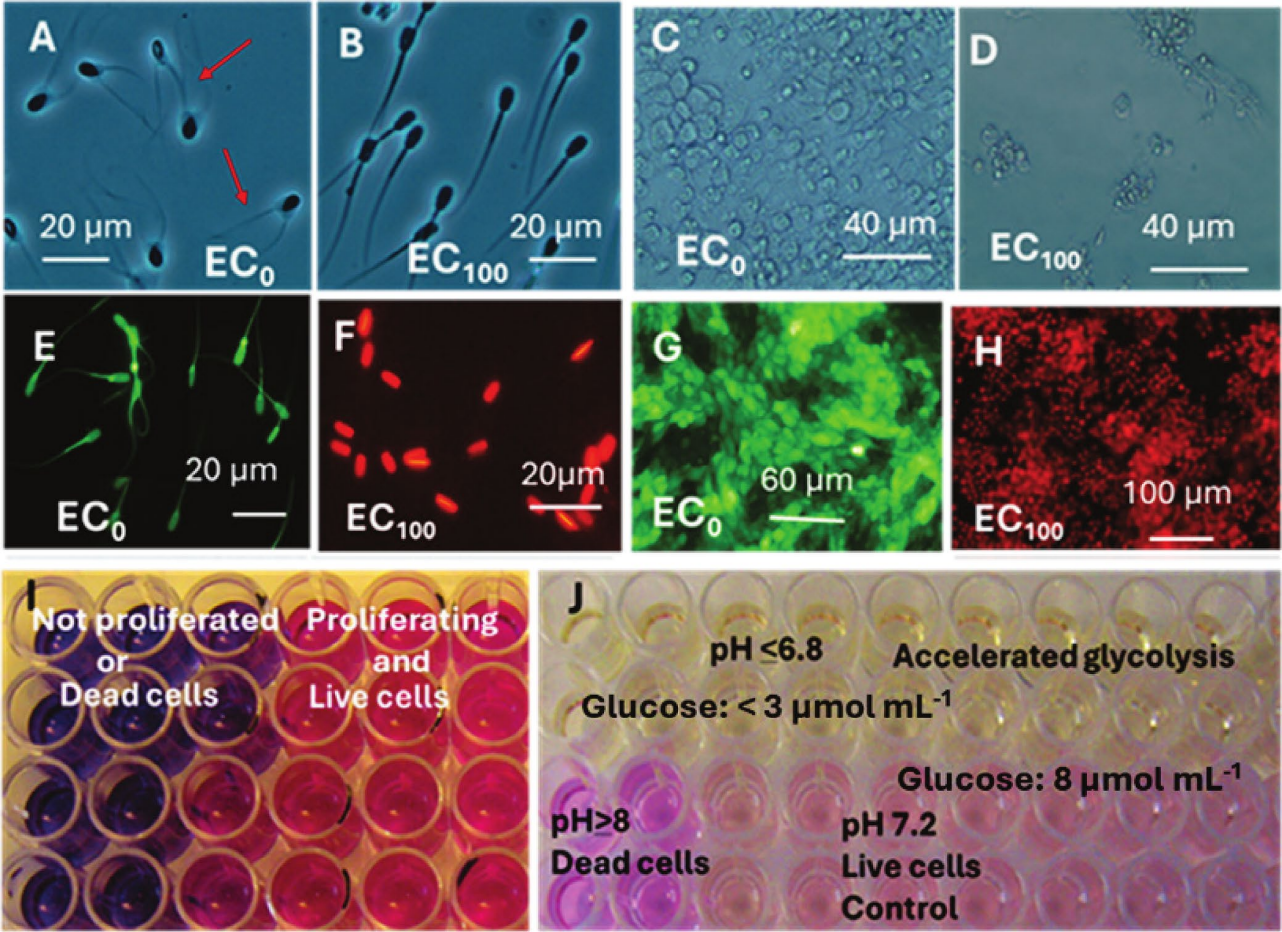
Endpoints of the BSMI, ICP, CD and MT bioassays illustrated as microscopic and metabolic readouts. The BSMI assay distinguishes rapidly swimming sperm cells (A) from paralysed ones (B). The ICP assay distinguishes proliferating somatic cells (C) from inactivated cells (D). The CD assay distinguishes live green‐fluorescing cells (E, F) from red fluorescing dead cells (G, H). Cell death is confirmed by absence of resazurin reduction (I) and depleted glucose consumption, accompanied by pH > 8 and glucose ≥ 12 μmol mL^−1^ (J). The MT assay measures mitochondrial dysfunction in somatic cells by accelerated glucose consumption and acidosis (J).

Values obtained for the inhibited motility in boar spermatozoa exposed to toxic substances were determined by visual inspection in a phase contrast microscope. Rapidly motile sperm cells and non‐motile or slowly motile sperm cells are visualized in Figure 3 Panels A and B. Iinhibition of sperm motility when exposed to certain toxins was confirmed from digital micrographic videos using a custom MATLAB script (Castagnoli et al. 2018). The EC_50_ concentration was defined as the concentration of extracted substance provoking a 50% decrease in the amount of rapid motile sperm cells. The positive control was a 10 mg mL^−1^ aqueous solution of triclosan, and tested concentrations were two‐fold dilutions between 1 to 10 μg mL^−1^.

#### 2.7.2 CD: Cell Death Assay Measures Decline in Cell Viability

Cell death was detected as loss of the plasma membrane integrity (Figure 3, Panels E, F and G, H) or/and as loss of resazurin reduction indicating depletion of cellular NADH (Figure 3, Panel I). Detailed protocols for microscopic assessment of cell death by live‐dead staining of sperm cells are described in Hitikka et al. (2024). In somatic cells, cell death was recorded in cells exposed as a monolayer (grown for 2 d) as permeability to propidium iodide, depletion of resazurin reduction, and glucose consumption (Figure 3 Panels G–J). EC_50_ was defined as the concentration depleting resazurin reduction and glucose consumption by ≥ 50% compared to that of the control. The positive control was triclosan in concentrations of 5–100 μg mL^−1^.

#### 2.7.3 ICP: Inhibition of Cell Proliferation Assay in Somatic Cell Lines, FFL and PK‐15 Cells (Feline Fetus Lung Cells and Porcine Kidney Cells)

Inhibition of cell proliferation was visualized in phase contrast microscope and confirmed by fluorometric readouts of resazurin reduction indicating depletion of cellular NADH. Proliferating and cytostatic cells are shown in Figure 3, Panels C and D. Protocols for the microscopic and fluorometric methods are described in Hitikka et al. (2024). EC_50_ concentration was calculated as the concentration of dry solids provoking a 50% decrease in emitted fluorescence of resorufin, the reduced form of resazurin compared to the control cells (Figure 3 Panel J) The positive control was triclosan, in tested concentrations of two‐fold dilutions between 4 and 16 μg mL^−1^.

#### 2.7.4 MT: Mitochondrial Toxicity Assay

Mitochondrial dysfunction in somatic cell lines was indicated by acceleration of glucose consumption and acidification of the growth medium from 7.2 to ≤ 6.8. EC_50_ was defined as the concentration of test substances where the concentration of glucose in the growth medium was ≤ 50% compared to that of the control. The end point for acidification coincided with the end point for depletion of glucose in the growth medium (Figure 3 Panel J). The protocol for the test estimating acceleration of glycolysis is described in Hitikka et al. (2024). The positive control was triclosan in concentrations between 0.5–5 μg mL^−1^. The endpoints of the four bioassays BSMI, CD, ICP and MT assays are presented in Figure 3.

### 2.8 Toxicity Profiles Based on Relationships Between EC_50_ Endpoints in the Four Bioassays

To avoid confusion, we distinguish between assay controls and model toxins used for profile classification. Triclosan and the biomass dispersal of cereulide‐producing *B. cereus* NC7041 were used as assay‐performance controls whereas alamethicin, surfactin, valinomycin, antimycin A, chaetoglobosin A and actinomycin D were used as reference compounds to define toxicity profiles I–IV.

The EC_50_ concentrations of bacterial extracts in the four bioassays were compared, and the extracts were classified into toxicity profiles I‐IV according to their response patterns. Positive responses (+) in the assays were defined as EC_50_ values ≤ 50 ug mL^−1^ for exposed boar sperm and < 100 ug mL^−1^ for somatic cells. Negative responses (−) were defined as EC_50_ values ≥ 20× greater than the EC_50_ values in the most sensitive bioassays. Commercial reference toxins used as positive controls and model compounds for the four toxicity profiles are shown in Table 1. Toxicity profile I: Model toxins were potassium channel forming alamethicin and the biosurfactant surfactin. Toxicity profile II: Model toxins were the potassium carrier ionophore valinomycin and the mitochondrial electron transport chain inhibitor antimycin A. Toxicity profile III: Model toxin was the cellular glucose transport inhibiting chaetoglobosin A. Toxin profile IV: Model toxins were the cytostatic RNA synthesis inhibiting Actinomycin D (Kleeff et al. 2000) and protein synthesis inhibiting sterigmatocystin.

### 2.9 Chemical Identification of the Bioactive Molecules

Methanol soluble substances from bacterial biomass were extracted into methanol, diluted in water (50%–90% methanol) and fractioned by a SEP‐Pak C18 cartridge (Waters Co Milford USA) and finally diluted in methanol. The sperm toxic Sepak fractions were further fractionated by reverse‐phase HPLC (RP‐HPLC, Smart Pharmacia Biotech Sweden). The fractions found active in the BSMI assay were soluble in 90%–100% methanol. After Chromatographic separation and purification, and the mass ions m/z of the sperm toxic substances were identified by the mass spectra (MS and MS/MS) analysis using API 300 triple quadrupole mass spectrometer (Perkin‐Elmer Sciex Instruments Thornhill Ontario Canada), ESI‐TQ, ESI‐IT with Escuire 2000 ion trap mass spectrometer (Burker Daltonik, Bremen Germany) and Matrix Assisted Laser Desorption/Ionization Time—Flight(MALDI‐TOF) with a BIFLEX MS (Burker‐Franzen Analytik, Bremen Germany). Amino acid composition and quantitation of the toxic substances were analysed by RP‐HPLC‐UV, or HPLC‐MS Agilent Series1100 HPLC (Wilmington Delaware USA). The purification, characterization and identification of the peptides are described in Andersson et al. (1998); Mikkola et al. (2007); Madslien et al. (2013); Mikkola et al. (2019).

### 2.10 Testing Antibacterial Effects of *B. amyloliquefaciens* and *B. cereus* Strains

To test heat stability and methanol solubility of extracted substances of selected strains and thereby exclude protein toxins, plate‐grown (TSA at 22°C for 1–2 days) biomass of amylosin, iturin, surfactin, fengysin‐producing *B. amyloliquefaciens* strains, cereulide‐producing *B. cereus* strains, and selected reference strains was extracted with hot methanol (60°C–80°C). The procedures for exposure and measurements of inhibition of growth of the target strains in TSB (Tryptic soy broth, Scharlab SL, Barcelona, Spain) are described in Rasimus‐Sahari et al. (2015). Briefly, the growth of the target strain after exposure was measured by turbidity measurements (Klett‐Summerson turbidometer, filter 520), confirmed by viable counting on TSA.

### 2.11 Testing Antifungal Activity of Biomasses Co‐Streaked on TSA Plates

The growth inhibitory effects were tested by co‐culturing biomass (20 mg) of the test strains and the target strains streaked on TSA plates for 10 days at 20°C ± 2°C.

### 2.12 Microbial Strains

Microbial strains were deposited in and retrieved from the Microbial Domain Biological Resource Centre (HAMBI), University of Helsinki, Finalnd; Deutsche Sammlung von Mikroorganismen und Zellkulturen (DSMZ), Braunsweig, Germany; Nagoya City Public Health Institute, Nagoya, Japan (NC) and the American Type Culture Collection (ATCC); and the authors' own collection. The strains are summarized in Table S2. Bacterial strains tested for growth inhibition of microbial strains used as target strains and in Table S4. Identification of the 108 toxigenic indoor strains, their separation into four toxicity profiles and identified toxins.

### 2.13 Reagents and Supplies

Cereulide, amylosin, lichenysin, and fusarcidins were purified from bacterial biomass of producer strains as described in Andersson et al. (1998), Mikkola et al. (2007) and Mikkola et al. (2019). Valinomycin, antimycin A, actinomycin D, surfactin, sterigmatocystin, and chaetoglobosin A, and the biocide triclosan were from Sigma‐Aldrich (St. Louis, MO, USA). Fengycin and iturin were from Aibi Lipopeptides (Gembloux, Belgium). The other chemicals were of analytical grade and purchased from local suppliers.

## 3 Results

### 3.1 Indoor Bacterial Isolates Producing Bioactive Metabolites

Out of 650 bacterial colonies recovered from 19 buildings approximately 20% exhibited microbial antagonism and were positive for metabolites affecting mammalian cells (in the BSMI and/or ICP assays). Of these, 111 colonies were pure cultured and identified to genus level and as cryptic *Actinobacteria* as shown in Table 2.

**TABLE 2:** Phenotypic characterization to genus level of indoor isolates screened positive in the BSMI (Boar sperm motility inhibition assay) and/or the ICP (Inhibition of somatic cell‐proliferation assay).

	Growth		Morphology, sporangia	FAME^a^	Genus
	37°C	50°C	Position		
Thermophilic endospore forming bacteria (*n* = 57)					
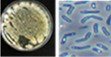	+	+	Central, ellipsoid, not swollen	Ante‐iso C15:0, C17:0	*Bacillus* (*n* = 36)
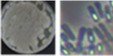	+	+	Terminal, ellipsoid, not swollen	Iso C13, C15:0	*Bacillus* (*n* = 21)
Mesophilic endospore forming bacteria (*n* = 11)					
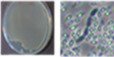	+	−	Central, ellipsoid, not swollen	Iso C14, C15:0	*Peribacillus* (*n* = 6)
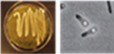	±	−	Terminal, spherical, swollen	Iso C16:0	*Lysinibacillus* (*n* = 3)
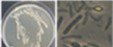	+	−	Terminal, ellipsoid swollen	Iso C 15:0	*Paenibacillus* (*n* = 2)
Mesophilic *Actinobacteria* (*n* = 30 + 13)					
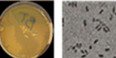	−	−	Small rods, no spores	Iso C16:0	*Microbacterium* (*n* = 3)
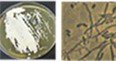	+	−	Branched mycelium, thermolabile spores in chains	Iso C16:0 TBSA acid^b^	*Nocardiopsis* (*n* = 4)
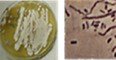	+	−	Branched mycelium, thermolabile spores in chains	Ante‐iso C15:0, C17:0	*Streptomyces* (*n* = 23)
Cryptic sporulating *Actinobacteria* (*n* = 13)					
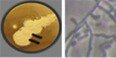			Branched mycelium thermolabile spores in chains		

^a^ Whole‐cell fatty acid methyl ester (FAME) analysis (Pirttijärvi et al. 1998).

^b^ Tuberculostearic acid, 10‐methyl‐C_18:0_ (Peltola, Andersson, Kämpfer, et al. 2001).

The 111 indoor strains represented four genera of endospore‐forming bacteria: *Bacillus*, *Peribacillus*, *Lysinibacillus*, *Paenibacillus*; two genera of *Actinobacteria*, *Streptomyces* and *Nocardiopsis* and in addition cryptic mesophilic sporulating *Actinobacteria* not identified to genus level.

### 3.2 Bacterial Species From Wet Building Materials and Indoor Dusts Producing Non‐Protein Toxins

Out of the 111 isolates screened positive in the BSMI and/or ICP assays, 95 bacterial strains were identified to species level and 13 strains remained classified as cryptic *Actinobacteria* as shown in Table 3. Three toxigenic isolates listed in Table 2 remained unclassified to species level.

**TABLE 3:** Bacterial strains (*n* = 108) screened positive in the BSMI assay and for antimicrobial activity (AM) their frequence in the 19 buildings and origin of isolates.

BSMI^a^ ICP^a^	AM^b^	Bacterial species/genera	Isolates (*n*)	Frequence in the 19 buildings	Origin of isolates		
					Material	Dust	Air
Thermophilic endospore forming bacterial strains (*n* = 55)							
+++	+++	*Bacillus amyloliquefaciens*	23	7/19	+	+	+
+	+	*B. licheniformis*	3	1/19	+	−	−
+	+	*B. subtilis*	5	1/19	+	−	−
+	+	*B. pumilus*	3	2/19	−	+	−
+++	+	*B. cereus*	21	6/19	+	+	−
Mesophilic endospore forming bacterial strains (*n* = 11)							
+	−	*Lysinibacillus* sp. close to *L. sphaericus*	3	1/19		+	−
+	++	*Paenibacillus polymyxa*	2	2/19		+	
+++	−	*Peribacillus simplex and* sp.	6	3/19		+	
Actinobacterial strains (*n* = 39 + 3)							
+++	+	*Streptomyces griseus*	14	4/19	+	+	+
+++	+	*Str. microflavus*	2	1/19			+
+++	+	*Str. albidoflavus*	6	3/19			+
+++	+	*Streptomyces* sp. close to *S. eheimensis and S. abikoensis*	1	1/19			+
+	−, +	*Nocardiopsis alba*	2	3/19	+		+
+	+	*Nocardiopsis exhalans*	1	1/19			+
(+)	+	Cryptic sporulating *Actinobacteria*	13	2/19	+	+	+
Non sporeforming actinobacterial strains (*n* = 3)							
++	+	*Microbacterium* sp. close to *M. esteroaromaticum*	3	1/19			+

^a^ BSMI (Boar sperm motility inhibition assay) +++ = positive after 5 min exposure. ++ positive after 1 day of exposure, in BSMI + positive after 2–4 days of exposure. ICP (Inhibition of cell proliferation assay) (+) = positive in ICP negative in BSMI.

^b^ AM (Antimicrobial activity) +++ = inhibition of growth observed against fungi and bacteria co‐growing on the isolation plates, ++ inhibiting fungi, + inhibiting bacteria – no inhibition of bacteria or fungi recorded.

Results in Table 3 show that strains of *Bacillus* sp., *Streptomyces* sp., *Nocardiopsis* sp., and unidentified cryptic sporulating *Actinobacteria* sp. were isolated from materials and dusts from 4 to 7 buildings, whereas strains of *Lysinibacillus* sp., *Peribacillus* sp., *Str. albidoflavus*, *Str. albidoviridis, Nocardiopsis exhalans*, and *Microbacterium* sp. were sporadically isolated from air or dust only. Results in Table 3 indicate that selecting antagonistic colonies and screening biomass suspensions from 108 indoor bacterial isolates antagonistic to co‐growing microbes revealed 15 different species and 13 cryptic isolates producing heat stable non‐protein compounds active against mammalian cells.

### 3.3 Lipopeptide Antibiotics and Unknown Non‐Protein Toxins Were Produced by Indoor Bacteria

The toxic substances identified as lipopeptide and depsipeptide antibiotics produced by selected indoor *Bacillus* sp., *Paenibacillus* sp., and *Streptomyces* sp. strains are shown in Table 4. *Bacillus amyloliquefaciens* strains produced four different lipopeptide antibiotics: amylosin, fengycin, iturin, and surfactin.

**TABLE 4:** Lipopeptide and depsipeptide antibiotics produced by indoor bacteria isolates, their molecular masses (Da), EC_50_ values in the BSMI assay (2d exposure at room temperature), producer species and reported biological activities.

Name and class of toxin	Da	EC_50_ μg mL^−1^	Bacterial species	Biological activity	References
Amylosin Lipopeptide	1197	0.03	*Bacillus amyloliquefaciens* HAMBI 2660	Channel forming ionophore	Mikkola et al. (2007)
Iturins Depsipeptide	1030–1044	6	*B. amyloliquefaciens* HAMBI 2660	Antibacterial	This study Rasimus‐Sahari et al. (2015)
Cereulide Depsipeptide	1152	< 0.001	*B.cereus* HAMBI 2454	Carrier ionophore	Mikkola et al. (1999), Andersson et al. (1998)
Fengycin A Depsipeptide	1464	40	*B. amyloliquefaciens* HAMBI 2660	Fungicidal	This study Rasimus‐Sahari et al. (2015)
Fusaricidins Lipopeptides	883–961	< 2	*Paenibacillus polymyxa* HAMBI 3103	Channel forming ionophore	Mikkola et al. (2019)
Lichenysins Lipopeptides	992–1034	< 8	*B. licheniformis* HAMBI 2463	Surfactant	Madslien et al. (2013)
Surfactins Lipopeptides	993–1035	< 10	*B. subtilis* HAMBI 2660	Surfactant	Madslien et al. (2013)
Valinomycin Depsipeptide	1110	≤ 0.004	*Streptomyces griseus* DSM 41750	Potassium carrier ionophore	Andersson et al. (1998)

### 3.4 Non‐Protein Toxins of Indoor Bacterial Strains Separated Into Four Toxicity Profiles

**FIGURE 4:**
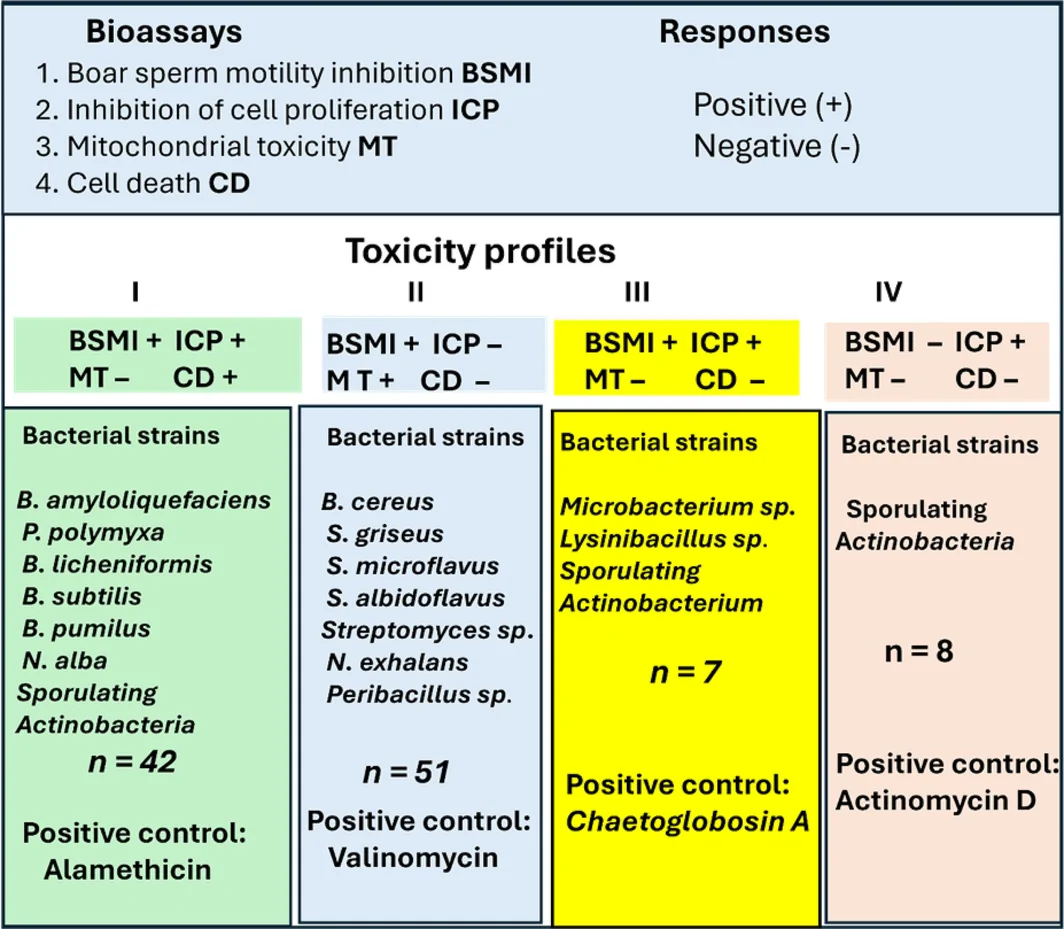
Toxicity profiles of methanol extracts of 108 indoor bacterial strains and control toxins. Substances extracted from bacterial biomass represented 4 toxicity profiles: lethal toxins (I) and sublethal toxins (II; III; IV).

Individual indoor bacterial strains identified to species level, their non‐protein toxins, toxicity endpoints and assignment to respective toxicity profiles are shown in Table S3. Toxicity profiles of heat stable methanol soluble substances extracted from 108 bacterial strains are in Table S4. Identification of the 108 toxigenic indoor strains, their separation into four toxicity profiles and identified toxins in the Supporting Information. The four toxicity profiles of the 108 indoor bacterial strains are summarized in Figure 4.

The biological activities recorded for extracts of the 108 bacterial strains and for the purified and commercial toxins used as positive controls are described below:

#### 3.4.1 Toxicity Profile I

Lethal toxins acting as membrane channel forming ionophores and surfactants were positive in the BSMI, CD, and ICP assays but negative in the MT assay. This profile was exhibited by the positive controls alamethicin and surfactin, by purified amylosin, surfactin/lichenysin, and fusaricidins. This toxicity profile was also exhibited by biomass extracts of strains of *Bacillus amyloliquefaciens* (*n* = 23), *B. licheniformis* (*n* = 3), *B. subtilis* (*n* = 5), *Paenibacillus polymyxa* (*n* = 2), *B. pumilus* (*n* = 3), *Nocardiopsis alba* (*n* = 2), and cryptic sporulating actinobacterial strains (*n* = 4).

#### 3.4.2 Toxicity Profile II

Sublethal carrier ionophores and mitochondrial toxins were positive in the BSMI and MT assays but negative in the CD and ICP assays. This profile was recorded for the positive controls valinomycin and antimycin A, and for purified cereulide and valinomycin. This profile was also exhibited by extracts of strains of *Bacillus cereus* (*n* = 21), *Streptomycetes griseus* (*n* = 14), *Str. microflavus* (*n* = 2), *Streptomyces* sp. (*n* = 1), *Streptomyces albidoflavus* (*n* = 6), *Peribacillus* sp. *close to P. simplex* (*n* = 6) and *Nocardiopsis exhalans* (*n* = 1).

#### 3.4.3 Toxicity Profile III

Sublethal cytostatic toxins inhibiting cellular glucose transport and cell proliferation. The positive control chaetoglobosin A was positive in the BSMI and ICP assays but negative in the CD and MT assays. This profile was exhibited by the *Lysinibacillus* sp. strains close to *L. sphaericus* (*n* = 3), *Microbacterium* sp. strains close to *M. esteraromaticum* sp. *(n = 3)*, and a cryptic sporulating actinobacterial strain (*n* = 1).

#### 3.4.4 Toxicity Profile IV

Sublethal cytostatic toxins were detected only in the ICP assay and werenegative in the BSMI, CD, and MT assays. This profile was represented by the cellular RNA synthesis inhibitor positive control, actinomycin D, the protein synthesis inhibitor sterigmatocystin, and by cryptic sporulating actinobacterial strains (*n* = 8).

Results in Tables 3 and 4 and Figure 4 show that strains that had provoked positive responses in the rapid screening tests for toxicity against mammalian cells produced lipopeptide antibiotics acting as surfactants, channel forming and mitochondrial‐toxic carrier ionophores, unidentified mitochondrial toxins and yet unknown cytostatic non‐protein toxins. Out of the 108 strains tested, mitochondrial toxins (Toxicity profile II) were produced by 47%; lethal cell death causing toxins (Toxicity profile I) were produced by 36%, respectively. Strains producing sublethal cytostatic toxins included in Toxicity profile III and IV represented only 14% of the tested strains. Mitochondrial toxin producing strains representing 7 species were the dominant toxin producers in indoor environments.

### 3.5 Occurrence of Valinomycin Producing *Streptomyces* sp. Strains in Various Environments

The results in Table 5 show that out of the 32 sporulating actinobacterial strains isolated from water‐damaged buildings, 17 were screened toxic, and out of these, 13 strains produced valinomycin. Out of the 190 strains isolated from various urban and rural closed spaces, food and feed and bedding materials, 49 screened positive in toxicity assays but none of the strains were valinomycin producers.

**TABLE 5:** Sperm toxicity and valinomycin production in 222 strains of sporulating actinobacteria.

		Sperm‐toxic strains		Non‐toxic strains	
		Valinomycin producer	Valinomycin non‐producer		
Water damaged urban buildings (*N*)^*^	Sample				
Day care centre (1)	Dust	2			4
Elementary school (1)	Air	1			2
Private dwelling (1)	Air	2	2		4
Private dwelling (1)	Dust	1	2		1
Bakery (1)	Screed	7			4
		Nr. of strains = 17		Nr. of strains = 15	
Dry urban buildings indoor dust (10)	Dust		4		36
Hay barn (3)	Hay		10		40
Hay barn (3)	Air		3		3
Stable (2)	Dust		4		6
Stable (1)	Silage		4		28
Stable (1)	Wood chips		13		20
Piggery (3)	Straw				5
Human food (4)	Cereals		4		2
Human food (2)	Potatoes		7		1
		Nr. of strains = 49		Nr. of strains = 141	

Abbreviation: N, number of samples investigated.

^*^ Number of samples investigated.

Valinomycin‐producing strains were the most frequently isolated toxigenic *Streptomyces* sp. strains from water‐damaged indoor environments. Valinomycin was produced by *Streptomyces griseus* , *S. microflavus* and one *Streptomyces* sp. strain, closely related to *S. abikoensis* . Results in Table 5 show that among 222 sporulating actinobacterial strains isolated from indoor environments, valinomycin producers were detected only in water damaged buildings.

### 3.6 Antimicrobial Activities of Amylosin Producing *Bacillus amyloliquefaciens* and Cereulide Producing *Bacillus cereus* Strains

The EC_50_ values for antibacterial and growth‐inhibitory substances produced by selected thermophilic *Bacillus* strains are listed in Table S5. Antibacterial and growth inhibiting effects of heat stable methanol extracts of selected *Bacillus* strains and pure toxins in Supporting Information.

Ssperm toxic methanol extracts of the amylosin‐producing *B. amyloliquefaciens* strains and pure amylosin inhibited the growth of all target bacteria, regardless of their growth temperatures and differences in cell wall structure. The target organisms included the acid‐fast *Mycobacterium murale* , and *Dietzia* sp., the Gram‐positive *Bacillus megaterium* , the Gram‐negative psychrotrophic *Sphingomonas aurantiaca* , and the obligate thermophilic Gram‐negative *Ureibacillus thermosphaericus* . In contrast, methanol extracts of the sperm toxic cereulide‐producing *B. cereus* strains and pure cereulide did not inhibit the growth of any target bacteria. Interestingly, the type strains *B. cereus* DSM 31 and *B. licheniformis* DSM13T, which were non‐toxic to sperm cells in the BSMI assay, inhibited growth of all tested target bacteria.

Antagonistic activity against fungi, *Neurospora crassa* and *Chaetomium globosum* strains exhibited by amylosin‐producing *Bacillus amyloliquefaciens* strains is presented in Table S6. Antifungal activity of *Bacillus amyloliquefaciens* and *Bacillus cereus* strains against *Neurospora* strains and an indoor *Chaetomium globosum* strain in Supporting Information. No antifungal activity was observed for the cereulide‐producing *Bacillus cereus* strains.

## 4 Discussion

Producer bacteria for most bioactive molecules have been isolated from outdoor sources such as plant rhizospheres, marine organisms or exotic or extreme environments (Chau et al. 2020; Li et al. 2025; Quinn et al. 2020). This article presents the indoor environment for the first time as a new, easily accessible and promising habitat for bio‐prospecting bacteria. Out of 650 tested indoor colonies, 108 strains produced heat‐stable methanol‐soluble non‐protein antibiotics and possible new bioactive substances. In this article methods for detection of non‐protein antibiotics from indoor environments are developed, reviewed, described and evaluated.

Nowadays, traditional methods such as plating assays, pure culturing, identification of isolated strains and screening for biological activity by bioassays have been replaced by genome mining methods. Modern genome mining analyses bacterial genomes to identify promising target genes coding for secondary metabolites, including potential antibiotics and drug candidates (Afni 2025; Foulston 2019; Selim et al. 2021). However, the first ionophoric peptide antibiotic valinomycin detected in 1950 and the chloride channel forming macrocyclic lactone‐ ivermectin, the ‘wonder drug’ from Japan, were discovered in the 1970s through traditional plating‐based isolation methods (Abbas et al. 2022; Crump and Ōmura 2011; Cerna‐Chávez et al. 2024; Huang et al. 2021; Quinn et al. 2020). After the 1970s only a few new molecules suitable for therapeutic drug development have been discovered (Huang et al. 2021; Quinn et al. 2020). The Reverse Alexander Fleming technique—RAFT, for detecting antagonistic colonies on nonselective media and the rapid screening method using single colony biomass suspension seemed effective in detecting a diversity of bioactive substance producing bacterial strains.

Instead of trying to create a standard method for isolation of bioactive metabolite producing bacteria, multiple cultivation conditions were applied in parallel: Cultivation direct on culture media enriches spore‐forming bacteria resistant to desiccation. Resuscitation protects sub‐lethally injured desiccation‐sensitive bacteria from osmotic stress (Andersson et al. 1995). We assumed that combining several non‐selective media, long incubation time and low temperature increased the desired diversity of the bacterial isolates. These methods may complement genome mining and may reveal unexpected drug candidates. Strains of endospore‐forming genera *Bacillus* sp., *Lysinibacillus* sp., *Paenibacillus* sp. *and Peribacillus* sp. as well as sporulating *Actinobacteria* sp., *Streptomyces* sp. and *Nocardiopsis* sp. are well‐known producers of bioactive molecules (Ahsan and Shimizu 2021; Chau et al. 2020; Lacey and Rutledge 2022; Manivasagan et al. 2013; Manetsberger et al. 2023; Mikkola et al. 2019; Peltola, Andersson, Kämpfer, et al. 2001; Sousa et al. 2025; Suominen et al. 2001; Taylor et al. 2005). Interestingly, the non‐spore‐forming *Microbacterium* sp. strains, which to our knowledge have not previously been reported as producers of such non‐protein bioactive substances, were also detected using a battery of new and traditional methods.

This study exploited differences among toxicity endpoints in ex vivo and in vitro assays to characterize the biological targets of the extracted substances. The small volume of boar spermatozoa, 22–25 μm^3^ (Du et al. 1994) may made them particularly sensitive to microbially produced non‐protein molecules that affected plasma membrane integrity, ion homeostasis, mitochondrial energy production, and cellular glucose transport. Comparisons across bioassays are also useful for identifying the most sensitive, simplest, or cheapest practical screening assays (Mohammadi‐Bardbori et al. 2008). Ionophoric toxins and toxins affecting mitochondrial function have previously yielded successful antiparasitic drugs (Abbas et al. 2022; Batiha et al. 2020; Crump and Ōmura 2011; Vicente‐Carrillo et al. 2015). Nonetheless, the division of the bacterial extracts and of the purified toxins into toxicity profiles based on endpoints in the battery of bioassays is very rough and speculative. The spectrum of biological effects in the extracts may be much larger than estimated based on the chosen positive control substances. Mitochondrial toxin producing strains representing 7 species and 47% out of the 108 tested bacterial strains seemed common in indoor environments.

Occurrence of antibiotic‐producing strains in wetted building materials, indoor dust, and air may be explained by their competitive advantage and indoor air and dust as a sink and a protected refuge. *B. amyloliquefaciens* , *B. subtilis, B. licheniformis, B. pumilus*, *B. cereus* , *Paenibacillus polymyxa*, *Streptomyces griseus* and *Nocardiopsis alba* strains colonized wet building materials (Table 3). In contrast, strains of *Lysinibacillus* sp., *Peribacillus* sp. *Str. albidoflavus*, *Str. albidoviridis, Nocardiopsis exhalans* and *Microbacterium* sp. were sporadically isolated from air or dust only (Table 3). These strains may represent bacteria of outdoor origin, or their indoor refuge material was not detected. Microbial antagonism explained occurrence of amylosin producers in wet building materials. Cereulide did not inhibit the growth of any of our target bacteria or fungi. Ladeuze et al. (2011) reported antifungal activity of cereulide and increased fitness of the producer strains in low‐potassium environments (Ekman et al. 2012) and in specific organic niches (Jenull et al. 2023).

There are bacteria found indoors that are rarely, if ever, isolated from the soil, rhizosphere or other natural environments typically targeted in bioprospecting studies (Afni 2025). Valinomycin‐producing strains of *Streptomyces* sp. were sought in many environments known to contain *Streptomyces* species, yet in this study they were isolated only from indoor dust, indoor air and building materials (Table 5). To our knowledge, *Bacillus* strains that simultaneously produce amylosin, fengycin, surfactin and iturins (Table 4) were not reported outside indoor built environments. This study demonstrated that indoor environments may represent accessible sources for bioprospecting bacteria producing ionophoric and mitochondriotoxic non‐protein molecules.

## Author Contributions

**Raimo Mikkola:** writing – review and editing. **Balázs Kakasi:** writing – review and editing. **Maria A. Andersson:** conceptualization, methodology, investigation, formal analysis. **Heidi Salonen:** writing – review and editing. **Szabolcs Nagy:** writing – review and editing.

## Conflicts of Interest

The authors declare no conflicts of interest.

## Supporting information


**Table S1:** The 23 samples collected from indoor environments of 19 buildings in Finland.
**Table S2:** Bacterial strains tested for growth inhibition of microbial strains used as target strains.
**Table S3:** Toxicity profiles of heat stable methanol soluble substances extracted from 108 bacterial strains. BSMI = boar sperm inhibition, CD = Cell death, ICP = inhibition of cell proliferation of somatic cell lines, MT = Mitochondrial toxicity.
**Table S4:** Identification of the 108 toxigenic indoor strains, their separation into four toxicity profiles and identified toxins.
**Table S5:** Antibacterial and growth inhibiting effects of heat stable methanol extracts of selected Bacillus strains and pure toxins.
**Table S6:** Antifungal activity of Bacillus amyloliquefaciens and Bacillus cereus strains against Neurospora strains and an indoor Chaetomium globosum strain. Antifungal activity recorded as antagonism on TSA plates was recorded as positive (+) or negative (−) by visible inhibition zones > 1 mm and invisible inhibition zones, < 0.05 mm, respectively.

## Data Availability

The data that supports the findings of this study are available in the Supporting Information of this article.
