# Supplementary material for: Bioprospecting Indoor Bacteria Producing Bioactive Metabolites
Source: Environ Microbiol Rep. 2026 Aug 23;18(4):e70402. doi: 10.1111/1758-2229.70402 (PMC13501004; doi:10.1111/1758-2229.70402)
Supplement: Supplementary file 1 [file EMI4-18-e70402-s001.docx]

**Supplementary Material**

**Table S1.** The 23 samples collected from indoor environments of 19 buildings in Finland.

| **Code** | **Sample*** | **Site and Space** | | **Building** | | **Location** | | **Growth media** | | **Resuscitated sample**** | |
| --- | --- | --- | --- | --- | --- | --- | --- | --- | --- | --- | --- |
| 1 | Screed | Wall, Storage | | Hospital | | Kuopio | | TSA | | + | |
| 2a | Insulation textile | Floor, Office | | University | | Helsinki | | TSA, MA | | + | |
| 2b | Settled dust | Shelf, Office | | University | | Helsinki | | TSA, MA | | - | |
| 3 | Insulation textile | Floor, Office | | Government  building | | Helsinki | | TSA, PCA | | - | |
| 4 | Insulation  paperboard | Wall, Livingroom | | Dwelling | | Helsinki | | TSA | | - | |
| 5 | Filler | Floor, Bathroom | | Dwelling | | Vantaa | | TSA | | + | |
| 6 | Screed | Wall, Workplace | | Factory | | Helsinki | | TSA | | + | |
| 7a | Liner | Wall Living room | | Day Care | | Helsinki | | TSA | | + | |
| 7b | Settled dust | Shelf, Living room | | Day Care | | Helsinki | | TSA | | + | |
| 8a | Bedding material | Floor, Piggery | | Weaned Piglet room | | Orimattila | TSA, PCA, MA | | +/- | |  |
| 8b | Dust | Floor, Piggery | | Weaned Piglet room | | Orimattila | TSA, PCA | | +/- | |  |
| 9 | Settled dust | Shelves, Office | | University | | Oulu | TSA, MA | | - | |  |
| 10 | Settled dust | Shelves, Livingroom | | Dwelling | | Lahti | TSA | | - | |  |
| 11 | Settled dust | Shelves, Livingroom | | Dwelling | Oulu | | TSA | | | - | |
| 12 | Settled dust | Shelves, Office | | Urban building | Oulu | | TSA, PCA | | | +/- | |
| 13 | Settled dust | Shelves, Storage | | Urban building | Kuopio | | TSA | | | - | |
| 14 | Settled dust | Frames, Storage | | Cow barn | Mäntsälä | | TSA | | | - | |
| **Air samples** | | | | | | | | | | | |
|  | **Sampling method** | | **Site and Space** | | | **Location** | | **Growth media** | |  | |
| 15a | Fall out plate | | School Classroom | | | Helsinki | | TSA, MA | | - | |
| 15b | Fall out plate | | School Classroom | | | Helsinki | | TSA, MA | | - | |
| 16 | Andersen Impactor | | Cow shed | | | Helsinki | | TSA, MA | | - | |
| 17 | Andersen impactor | | Dwelling, Living room | | | Espoo | | TSA | | - | |
| 18 | Andersen impactor | | School classroom | | | Helsinki | | TSA | | - | |
| 19 | Fall out plate | | Dwelling Living room | | | Espoo | | TSA | |  | |

***** Air and dusts were collected 1.5-2 m above floor level. ** Samples marked as (+) were resuscitated before plating, samples recorded as (–) were plated directly and samples recorded as (+/-) were plated both with and without resuscitation, respectively.

**Table S2**. Bacterial strains tested for growth inhibition of microbial strains used as target strains

| **Bacterial Test strains (Indoor isolates)** | | | | |
| --- | --- | --- | --- | --- |
|  | **Strain code^1^** | **Identified toxic metabolite** | **Origin**  **Sample code^1^** | **References** |
| *B. amyloliquefaciens* | HAMBI 2660 | Amylosin  Fengysin  Surfactin  Iturin | 2b | Rasimus et al., 2015  Mikkola et al., 2007 |
| *B. amyloliquefaciens* | HAMBI 2662 | Amylosin  Surfactin | 3 | Mikkola et al., 2007 |
| *B. amyloliquefaciens* | HAMBI 2663 | Amylosin  Surfactin | 3 | Mikkola et al., 2007 |
| *B. cereus* | HAMBI 2517 | Cereulide | 1 | Apetroaie et al., 2005 |
| *B. cereus* | HAMBI 2711 | Cereulide | 1 | Apetroaie et al., 2005 |
| *B. cereus* | LKT1/1 | Cereulide | 5 | Apetroaie et al., 2005 |
| **Bacterial Reference strains** | | | | |
| *B. amyloliquefaciens ^T^* | DSM 7 | Nontoxic |  | This study |
| *B. amyloliquefaciens* | HAMBI 2718 | Nontoxic |  | Rasimus-Sahari et al., 2015 |
| *B. amyloliquefaciens* | FZB42 | Nontoxic |  | Chau et al., 2020 |
| *B. cereus^T^* | ATCC14789 | Nontoxic |  | This study |
| *B.cereus* | HAMI 2450 | Cereulide |  | Rasimus et al., 2012 |
| *B.cereus* | NC7401 | Cereulide |  | This stydy |
| *B.liheniformis^T^* | DSM13 | Nontoxic |  | This study |
| *B. megaterium* | DSM17641 | Nontoxic | 4 | Rasimus-Sahari et al., 2015. |
| *B. mycoides* | ATCC 6462 | Nontoxic |  | This study |
| **Bacterial target strains** | | | | |
| *B. cereus* | HAMBI 2450 | Cereulide |  | Rasimus-Sahari et al., 2015. |
| *B. cereus* | ATCC 4342 | Nontoxic |  | Apetroaie et al., 2005 |
| *Dietzia* sp*.* | *MA147* | Nontoxic | 7a | Rasimus-Sahari 2016 |
| *Mycobacterium murale^T^* | DSM44340 | Nontoxic | 7a | Vuorio et al., 1999 |
| *Sphingomonas aurantiaca*^T^ | DSM147481 | Nontoxic |  | Busse et al., 2003 |
| *Ureibacillus thermosphaericus^T^* | DSM 113452 | Nontoxic |  | Andersson et al., 1995 |
| *Williamsia muralis*^T^ | DSM 44343 | Nontoxic | 7a | Kämpfer et al., 1999 |
| **Fungal target strains** | | | | |
| *Aspergillus niger* | T21 |  | 1 |  |
| *Chaetomium globosum* | HAMBI 3643 | Chaetoglobosin A | 1 | Salo et al., 2020 |
| *Nerospora crassa* | DSM 894 | Nontoxic |  | This study |
| *N.crassa* | DSM1247 | Nontoxic |  | This study |
| *N.crassa* | DSM 1258 | Nontoxic |  | This study |

^1^ Origin of Sample and Sample code in Table S1 in supplemental material.

***Table S3****. Toxicity profiles of heat stable methanol soluble substances extracted from 108 bacterial strains. BSMI= boar sperm inhibition, CD=Cell death, ICP= inhibition of cell proliferation of somatic cell lines, MT= Mitochondrial toxicity.*

| **Toxicity profile I: Toxins positive for the 3 assays BSMI, CDs and ICP. Negative in MT** | | | | | | | | | |
| --- | --- | --- | --- | --- | --- | --- | --- | --- | --- |
|  | | Toxicity assay responses +/-  (End points EC_50_ µg mL^-1^) | | | | | | |  |
| **Species**  **(nr. of isolates)** | **Model strain** | **BSMI** | | **CD** | **ICP** | | | **MT** | **References** |
| N=42 | | **+** | | **+** | **+** | | | **-** |  |
| *Bacillus amyloliquefaciens* (n=23) | HAMBI 2660  =19b | + (≤ 0.1) | | + (≤ 0.1) | + (2-3) | | | - None | This study  Mikkola et al., 2007 |
| *Paenibacillus. polymyxa*  (n=2) | HAMBI 3103 | + (4) | | + (12) | + (60) | | | - None | Mikkola et al., 2019 |
| *B. licheniformis* (n=3) | THG6 | + (40) | | + (40) | + (100) | | | - None | This sudy |
| *B. subtilis*  (n=5) | LL15 | + (30) | | + (30) | + (100) | | | - None | This study |
| *B. pumilus*  (n=3) | HAMBI 2666 | + (10) | | + (10) | + (50) | | | - None | This study |
| Spoulating *Actinobacteria* (n=3) | HS101 | 0.8 | | 3 | 30 | | | - None | This study |
| Sporulating *Actinobacteria* (n=1) | M31 | 4 | | 8 | 30 | | | - None | This study |
| *Nocardiopsis alba* (n=2) | DSM 44412 | 10 | | 30 | ni | | | NI | Peltola et al., 2000b |
|  |  | Toxicity assay responses +/-  (End points EC_50_ µg mL^-1^) | | | | | | |  |
| **Positive controls** | **Origin** | **BSMI** | | **CD** | **ICP** | | | **MT**** |  |
| Alamethicin* | SA* | 0,6 | | 0.6 | 8 | | | none |  |
| Surfactin* | SA* | 5 | | 5 | 20 | | | none |  |
| **Purified toxins** | **Producer strain** | | |  |  | | |  |  |
| Amylosin | HAMBI 2660 | + (0.03) | | + (0.03) | + (0.2) | | | - None | This study  Mikkola et al., 2007 |
| Fengycin* | HAMBI 2662 | >20 | | NI | NI | | | NI | This study |
| Iturin family Bacillomycin D | HAMBI 2662 | + (6) | | NI | NI | | | NI | This study |
| Fusarisidins | HAMBI 3103 | + (0.5) | | + (2) | + (10) | | | - None | Mikkola et al., 2019 |
| Lichenysin | THG6 | + (4-8) | | + (4-8) | + (20) | | | - None | This study |
| Surfactin | HAMBI 2662, LL15 | + (5-7) | | + (5-7) | + (20-30) | | | - None | Mikkola et al., 2007. This study |
| **Toxicity profile II: Toxins positive in the BSMI, negative in CD and ICP, positive in MT** | | | | | | | | | |
|  |  | Toxicity assay responses +/-  (End points EC_50_ µg mL^-1^) | | | | | | |  |
|  |  | **BSMI** | | **CD** | **ICP** | | | **MT** |  |
| N=51 |  | **+** | | **-** | **-** | | | **+** |  |
| *B. cereus* (n=21) | HAMBI 2711 | + (0.03) | | - (>50) | - (>10) | | | 0.05 | Andersson et al., 2005  This study |
| *Str. griseus*  (n=14) | DSM 41818 | + (0.05) | | - (>50) | - (>50) | | | 0.08 | Andersson et al., 1998. Andersson et al., 2005  This study |
| *Str.microflavus* (n=2) | DSM 3327 | + (0.04) | | - (>50) | - (>10) | | | 0.09 | This study |
| *Str. albidoflavus* (n=6) | DSM 41816 | + (0.1) | | - (>50) | - (>10) | | | NI | This study |
| *Peribacillus. simplex* and sp. (n=6) | DSM 13997 | + (0.1) | | - (>50) | - (>100) | | | NI | Peltola et al., 2001a  This study |
| *N. exhalans*  (n=1) | DSM 44407 | + (≤ 30) | | - (>100) | NI | | | NI | Peltola et al., 2001b |
| *Streptomyces* sp*.* (n=1) | DSM 41774 | | + (0.05) | - (>100) | NI | | | NI | Peltola et al., 2001a |
|  |  | Toxicity assay responses +/-  (End points EC_50_ µg mL^-1^) | | | | | | |  |
| **Positive controls** | **Origin** | **BSMI** | | **CD** | **ICP** | | | **MT** |  |
| Valinomycin* | SA* | + (<0.003 | | - (>1)- | - (>1) | + (< 0.003) | | | Salo et al., 2020 |
| Antimycin A* | SA* | + (0.005) | | - (>1) | - (>1) | | | 0.006 | This study |
| **Purified toxins** | **Producer strain** | | |  |  | | |  |  |
| Cereulide | HAMBI 2711 | + (0.0005) | | - (>1) | - (>1) | | + (0.0004) | | This study |
| Valinomycin | DSM 41818  DSM 3327 | + (0.003) | | - (>1) | - (>1) | | | 0.005 | Salo et al., 2020 |
|  | | | | | | | | | |
| **Toxicity profile III: Toxins positive for the two assays BSMI and ICP, negative in CD, MT** | | | | | | | | | |
|  |  | Toxicity assay responses +/-  (End points EC_50_ µg mL^-1^) | | | | | | |  |
|  |  | **BSMI** | | **CD** | **ICP** | | | **MT** |  |
| N=7 |  | **+** | | **-** | **+** | | | **-** |  |
| *L. sphaericus*  (n=3) | HAMBI 3319 | + (2) | | - (>100) | + (30) | | | - None | This study |
| *Microbacterium* sp*. (n=3)* | HAMB I3329 | + (1) | | - (>100) | + (≤ 30) | | | - None | This study |
| Sporulating *Actinobacteria* | M 23 | + (12) | | - (>50) | + (< 10) | | | - None | This study |
|  |  | Toxicity assay responses +/-  (End points EC_50_ µg mL^-1^) | | | | | | |  |
| **Positive control** | **Origin** | **BSMI** | | **CD** | **ICP** | | | **MT** |  |
| Chaetoglobosin A* A* | SA* | + (1) | | - (> 20) | + (3) | | | - None | Salo et al., 2020 |
|  |  |  |  |  |  |  |  |  |  |
|  |  |  |  |  |  |  |  |  |  |
| **Toxicity profile IV: Toxins positive for the ICP assay only.** | | | | | | | | | |
|  |  | Toxicity assay responses +/-  (End points EC_50_ µg mL^-1^) | | | | | | |  |
|  |  | **BSMI** | | **CD** | **ICP** | | | **MT** |  |
| N=8 |  | **-** | | **-** | **+** | | | **-** |  |
| Sporulating *Actinobacteria* (n=8) | M25 | - (>100) | | - (>100) | + (10) | | | - None | This study |
|  |  | Toxicity assay responses +/-  (End points EC_50_ µg mL^-1^) | | | | | | |  |
| **Positive controls** | **Origin** | **BSMI** | | **CD** | **ICP** | | | **MT** |  |
| Actinomycin D* | SA* | - (>20) | | - (>100) | + (< 0.2) | | | - None | This study |
| Sterigmatocystin* | SA* | - (>20) | | - (30) | + (0.3) | | | - None | Salo 2020  This study |
| **Positive control for assay calibration** | | | | | | | | | |
| Triclosan* | SA* | + (1) | | 25-50 | 10 | | | 5-10 |  |

* Pure substances used in bioassays were from Sigma-Aldrich

*Table S4. Identification of the 108 toxigenic indoor strains, their separation into four toxicity profiles and identified toxins.*

|  |  | | **Origin (building)** | | **Identification** | | |  | |
| --- | --- | --- | --- | --- | --- | --- | --- | --- | --- |
| **Lab**  **code** | **Collection**  **code** | | **Sample**  **code** | **Source** | **FAME** | **Ribo**  **type** | ***16S rDNA***  **seq.** | **References** | |
| **Toxicity profile I: Strains producing lethal toxins depleting plasma membrane integrity** | | | | | | | | | |
| **Amylosin producing *Bacillus amyloliquefaciens* (n=23)** | | | | | | | | | |
| 1/117 | Hambi 2662 | | 3 | Textile | + | + | + | Mikkola et al., 2004 | |
| 2/117 | Hambi 2664 | | 3 | Textile | + | + | + | Mikkola et al., 2004 | |
| 5/117 | Hambi 2661 | | 3 | Textile | + | + |  | Mikkola et al., 2004 | |
| LL19b | Hambi 2660 | | 2b | Dust | + | + |  | Mikkola et al., 2004 | |
| 201 |  | | 2b | Dust | + |  |  | Andersson et al., 2005 | |
| 204 |  | | 2b | Dust | + |  |  | This study | |
| 205 |  | | 2b | Dust | + |  |  | This study | |
| 206 |  | | 2b | Dust | + |  |  | This study | |
| 200 |  | | 2b | Dust | + |  |  | This study | |
| 205 |  | | 2b | Dust | + |  |  | This study | |
| 206 |  | | 2b | Textile | + |  |  | This study | |
| 207 |  | | 2b | Textile | + |  |  | This study | |
| 208 |  | | 2b | Dust | + |  |  | This study | |
| 210 |  | | 2b | Textile | + |  |  | This study | |
| L15b |  | | 2a | Textile | + |  |  | This study | |
| L19b |  | | 2a | Textile | + |  |  | This study | |
| 2.14 | Hambi 2663 | | 5 | Material | + | + |  | This study | |
| TAV1 |  | | 6 | Dust | + |  |  | Andersson et al., 2010 | |
| TAV5 |  | | 6 | Dust | + |  |  | Andersson et al., 2010 | |
| TAV16 |  | | 6 | Dust | + |  |  | Andersson et al., 2010 | |
| 102 |  | | 12 | Dust | + |  |  | This study | |
| O11 | Hambi 3321 | | 11b | Dust | + |  | + | This study | |
| O14 | Hambi 3320 | | 11b | Dust | + |  | + | This study | |
| **Lichenysin producing *Bacillus licheniformis* (n= 3)** | | | | | | | | | |
| 4/thg |  | | 5 | Liner | + |  |  | This study | |
| 5/thg |  | | 5 | Liner | + |  |  | This study | |
| 6/thg |  | | 5 | Liner | + |  |  | This study | |
| **Surfactin producing *Bacillus subtilis* (n=5)** | | | | |  |  |  |  | |
| LL14 |  | | 2a | Textile | + |  |  | This study | |
| LL15 |  | | 2a | Textile | + |  |  | This study | |
| LL1 |  | | 2a | Dust | + |  |  | This study | |
| LL6 |  | | 2a | Dust | + |  |  | This study | |
| LL9 |  | | 2a | Dust |  |  |  | This study | |
| **Fusarisisdins producing *Paenibacillus polymyxa* strains (n=2)** | | | | | | | | | |
| RS10 | HAMBI 3013 | |  | Dust | + | + | + | Mikkola et al., 2019 | |
| 1/Sim | HAMBI 3014 | |  | Dust | + | + | + | Mikkola et al., 2019 | |
| **Unknown toxins producing *Bacillus pumilus* (n=3)** | | | | | | | | | |
| ES20 | DSM 13835 | | 15 | Air | + | + | + | Peltola et al., 2001a | |
| 149/9 | Hambi 2666 | | 12 | Dust | + |  |  | This study | |
| 135/9 |  | | 12 | Dust | + |  |  | This study | |
| **Unknown toxin producing *Nocardiopsis alba* strains (n=2)** | | | | | | | | | |
| 704a | DSM 44412 | | 18 | air | + |  | + | Peltola et al., 2001b | |
| 123 | DSM 43377 | | 7a | liner | + |  | + | Peltola et al., 2001b | |
| **Unknown toxin producing sporulating *Actinobacteria* (n=4)** | | | | | | | | | |
| HS101 |  | | 14b | Air |  |  |  | This study | |
| HS102 |  | | 14b | Air |  |  |  | This study | |
| HS103 |  | | 14b | Air |  |  |  | This study | |
| M31 |  | | 8a | Materia |  |  |  | This study | |
| **Toxicity profile II: Strains producing sublethal mitochondrial toxins** | | | | | | | | | |
| **Cereulide producing *Bacillus cereus* (n=21)** | | | | |  |  |  |  | |
| LKT1/1 | |  | 5 | Filler | + | + | + | Apetroaie et al., 2005 | |
| 35H | Hambi 2668 | | 5 | Filler | + | + | + | This study | |
| 2/pk1  =1/n1 | Hambi 2513 | | 1 | Screed | + | + | + | This study | |
| 2/PK1  =2/n1 | Hambi 2514 | | 1 | Screed | + | + | + | This study | |
| 3/pk1  =3/n1 | Hambi 2515 | | 1 | Screed | + | + | + | This study | |
| 4/pk1  =4/ni | Hambi  2516 | | 1 | Screed | + | + | + | This study | |
| 5/n2 |  | | 1 | Screed | + | + | + | This study | |
| 6/n2 |  | | 1 | Screed | + | + | + | This study | |
| 7/n2  =7/pk2 | Hambi2711 | | 1 | Screed | + | + | + | Andersson et al., 2005 | |
| 8/n2 |  | | 1 | Screed | + | + | + | This study | |
| 9/n2  =9/pk1 | Hambi 2517 | | 1 | Screed | + | + | + | This study | |
| 4.3 |  | | 1 | Screed | + | + | + | This study | |
| TAV 5 | HAMBI 3326 | | 9 | Dust | + | + | + | Andersson et al., 2010 | |
| TAV17 |  | | 9 | Dust | + |  | + | Andersson et al., 2010 | |
| TAV19 |  | | 9 | Dust | + |  | + | Andersson et al., 2010 | |
| TAV20 |  | | 9 | Dust | + |  | + | Andersson et al., 2010 | |
| TAV33b | |  | 9 | Dust | + |  | + | Andersson et al., 2010 | |
| TAV35 |  | | 9 | Dust | + | + | + | Andersson et al., 2010 | |
| TAV37 |  | | 9 | Dust | + | + | + | Andersson et al., 2010 | |
| 141/9 |  | | 10 | Dust |  |  |  | This study | |
| MO32 |  | | 8b | Bedding Material |  |  |  | This study | |
| **Unknown toxin producing *Peribacillus simplex* and *Peribacillus* sp*.* (n=6)** | | | | | | | |  | |
| ES21 | DSM 13997 | | 15 | Air | + | + | + | Peltola et al., 2001a | |
| S100 |  | | 2b | Dust | + | + | + | This study | |
| DTA |  | | 11a | Dust | + |  |  | This study | |
| DTB |  | | 11a | Dust | + |  | + | This study | |
| DT3 |  | | 11a | Dust | + |  |  | This study | |
| DT4 |  | | 11a | Dust | + |  |  | This study | |
| **Valinomycin producing *Streptomyces griseus/ griseus/anulatus* , and *Streptomyces* sp.(n=14)** | | | | | | | | | |
| 2/ppi | DSM 41749 | | 7b | Dust | + | + | + | Andersson et al., 1998 |  |
| 8/ppi | DSM 41750 | | 7b | Dust |  |  |  | Andersson et al., 1998 |  |
| 10/ppi | DSM 41751 | | 7b | Dust |  |  |  | Andersson et al., 1998 |  |
| 5AL |  | | 6 | Screed |  |  |  | This study |  |
| 6AL | DSM 41818 | | 6 | Screed | + | + | + | Andersson et al., 2005 |  |
| 7AL |  | | 6 | Screed |  |  |  | This study |  |
| 8AL |  | | 6 | Screed |  |  |  | This study |  |
| 9AL |  | | 6 | Screed |  |  |  | This study |  |
| 10AL | DSM 41817 | | 6 | Screed | + | + | + | This study |  |
| 1AL |  | | 6 | Screed |  |  |  | This study |  |
| 12AL |  | | 6 | Screed |  |  |  | This study |  |
| 1/k | DSM 41748 | | 14a | Air | + | + | + | Andersson et al., 1998 |  |
| ES9* | DSM 41772 | | 15 | Air | + | + | + | Peltola et al., 2001a |  |
| ES14* | DSM 41773 | | 15 | Air | + | + | + | Peltola et al., 2001a |  |
| **Valinomycin like toxin producing *Streptomyces sp*. stain close to *S. abikoensis* and *S. ehimensis*.** | | | | | | | | | |
| ES16* | DSM 41774 | | 15 | Air | + | + | + | Peltola et al., 2001a | |
| **Valinomycin producing *Streptomyces microflavus* (n=2)** | | | | | | | | | |
| MP2 | Hambi 3327 | | 15 | Air |  |  |  | This study | |
| MP10 |  | | 15 | Air |  |  |  | This study | |
| **Antimycin like toxin sproducing *Streptomyces albidoflavus* (n=6)** | | | | | | | | | |
| Fav2 |  | | 10 | Air | + | + | + | This study | |
| N15 | DSM 41814 | | 13b | Air | + | + | + | This study | |
| N16 | DSM 41813 | | 13b | Air | + | + | + | This study | |
| N19 | DSM 41815 | | 13b | Air | + | + | + | This study | |
| N20 | DSM 41816 | | 13b | Air | + | + | + | This study | |
| N27 | DSM 41812 | | 13b | Air | + | + | + | This study | |
| **Unknown toxin producing *Nocardiopsis exhalans* (n=1)** | | | | | | | |  | |
| ES10.1 | DSM 44407 | | 15 | Air | + | + | + | Peltola et al., 2001a | |
| **Toxicity profile III: Strains producing cytostatic toxins inhibiting boar sperm motility** | | | | | | | | | |
| ***Lysinibacillus* strains, close to *L. sphaericus*, producing unknown toxins (n=3)** | | | | | | | | | |
| O21 | HAMBI 3319 | | 11b | Dust | + | + | + | This study | |
| O22 |  | | 11b | Dust | + |  |  | This study | |
| O23 |  | | 11b | Dust | + |  |  | This study | |
| ***Microbacterium* sp. strains, close to *M.esteroaromaticum* producing unknown toxins (n=3)** | | | | | | | | | |
| HS-4 | Hambi  3329 | | 14a | Air | + |  | + | This study | |
| HS-5 |  | | 14a | Air | + |  |  | This study | |
| HS-16 | Hambi  3331 | | 14a | Air | + |  | + | This study | |
| **Unknown toxin producing sporulating *Actinobacteria* (n=1)** | | | | | | | | | |
| MO23 |  | | 8a |  |  |  |  | This study | |
| **Toxicityprofile IV Strains producing cytostatic toxins** | | | | | | | | | |
| **Sporulating strains of *Actinobacteria* producing unknown toxins (n=8)** | | | | | | | | | |
| 01/02 |  | | 19 | Air | + |  |  | This study | |
| M25 |  | | 8a | Material |  |  |  | This study | |
| C1 |  | | 8a | Material |  |  |  | This study | |
| C4 |  | | 8a | Material |  |  |  | This study | |
| C11 |  | | 8b | Dust |  |  |  | This study | |
| C22 |  | | 8b | Dust |  |  |  | This study | |
| C8 |  | | 8b | Dust |  |  |  | This study | |
| C53 |  | | 8b | Dust |  |  |  | This study | |

**Table S5.** Antibacterial and growth inhibiting effects of heat stable methanol extracts of selected *Bacillus* strains and pure toxins.

|  | | | | Toxicity endpoints of methanol soluble substances (EC_50_ µg ml^-1^) | | | | | | | | | | |  |
| --- | --- | --- | --- | --- | --- | --- | --- | --- | --- | --- | --- | --- | --- | --- | --- |
|  | | Boar sperm | | | | | Exposed bacterial strains, incubation temperature* | | | | | | | |  |
|  |  | | BSMI 0.5 h | | | *Mycobacterium murale^T^*  DSM 44340 | | | *Bacillus megaterium*  DSM 17641 | *Dietzia sp.* MA147 | *Sphingomonas aurantiaca*  *DSM 14748* | | | *Ureibacillus thermosphaericus* DSM 11345 | |
| Test strains | | | | 22^o^C | | | 28^o^C | 28 °C | | 28 °C | | 18 °C | 60 °C | |  |
| *B. amyloliquefaciens* strains producing amylosin, surfactin, iturin and fengysin | | | | | | | | | | | | |  | |  |
| HAMBI 2662 | | | | 50 | 25 | | | 50 | | 50 | | 50 | 100 | |  |
| HAMBI 2660 | | | | 25 | 50 | | | 25 | | 25 | | 50 | 100 | |  |
| HAMBI 2663 | | | | 25 | 50 | | | 25 | | 25 | | 25 | 100 | |  |
| *B. cereus* strains producing cereulide | | | | | | | | | | | | |  | |  |
| HAMBI 2711 | | | | < 0.3 | >100 | | | >100 | | >100 | | >100 | >100 | |  |
| HAMBI 2667 | | | | < 0.3 | >100 | | | >100 | | >100 | | >100 | >100 | |  |
| HAMBI 3326 | | | | < 0.3 | >100 | | | >100 | | >100 | | >100 | >100 | |  |
| Reference bacterial strains nontoxic in the BSMI assay | | | | | | | | | | | | | | |  |
| *B. amyloliquefaciens* | | | | | | | |  | |  | |  |  | |  |
| DSM 7^T^ | | | | > 250 | >100 | | | >100 | | >100 | | >100 | >100 | |  |
| HAMBI 2718 | | | | > 250 | >100 | | | >100 | | >100 | |  |  | |  |
| FZB42 | | | | > 250 | >100 | | | >100 | | >100 | |  |  | |  |
| MO1 | | | | > 250 | >100 | | | >100 | | >100 | |  |  | |  |
| *B. cereus* | | | | | | | | | | | | | | |  |
| DSM31^T^ | | | | > 250 |  | | | 1 | | 0.25 | | <25 | <25 | |  |
| *B.mycoides* | | | |  |  | | |  | |  | |  |  | |  |
| ATCC 6462^T^ | | | | >100 | >100 | | | >100 | | >100 | |  |  | |  |
| *B. licheniformis* | | | | | | | |  | |  | |  |  | |  |
| DSM 13^T^ | | | | >100 |  | | | 30 | |  | |  |  | |  |
| Positive controls (pure substances) | | | | | | | |  | |  | |  |  | |  |
| Iturin | | | | 100 |  | | | 6 | | 10 | |  |  | |  |
| Fengycin | | | | 100 |  | | | > 40 | |  | |  |  | |  |
| Surfactin | | | | 100 |  | | | >50 | | 10 | |  |  | |  |
| Amylosin | | | | 0.1 | < 0.1 | | | 0.06 | | 0.06 | | 0.2 |  | |  |
| Cereulide | | | | 0.002 |  | | | >100 | | >100 | | >100 | >100 | |  |

* Details of the strains used are given in **Table S2**

***Table S6****. Antifungal activity of Bacillus amyloliquefaciens and Bacillus cereus strains against Neurospora strains and an indoor Chaetomium globosum strain. Antifungal activity recorded as antagonism on TSA plates was recorded as positive (+) or negative (-) by visible inhibition zones ≥ 1 mm and invisible inhibition zones, < 0.05 mm, respectively.*

|  | **Target organisms** | | | | |
| --- | --- | --- | --- | --- | --- |
|  |  | **Exposed fungal strains** | | | |
|  | **Boar**  **sperm** | ***N. crassa***  **DSM 894** | ***N.crassa***  **DSM1247** | ***N.crassa***  **DSM 1258** | ***C.globosum***  **HAMBI 3643** |
| ***Bacillus amyloliquefaciens* strains** | | | | | |
| HAMBI 2662 | + | + | + | + | + |
| HAMBI 2660 | + | + | + | + | + |
| HAMBI 2663 | **+** | **+** | **+** | **+** | + |
| ***Bacillus cereus* strains** | | | | | |
| HAMBI 2711 | + | - | - | - | - |
| HAMBI 2667 | + | - | - | - | - |

References

1. Apetroaie, C., M. A. Andersson, C. Sproer, I. Tsitko, R. Shaheen, E. L. Jääskeläinen, L. M. Wijnands, R. Heikkila, and M. S. Salkinoja-Salonen. 2005. Cereulide-producing strains of Bacillus cereus show diversity. Archives of Microbiology 184 (3):141–51. doi:10.1007/s00203-005-0032-1.
2. Andersson, M.A., Jääskeläinen, E.-L., Teplova, V., Veijalainen, P., Apetroaie, C., Hoornstra, D., Kroppenstedt, R.M., and Salkinoja-Salonen, M. (2005) Indoor bacilli and streptomycetes produce substances toxic to mammalian cells. In Bioaerosols, Fungi, Bacteria, Mycotoxins and Human Health: Pathophysiology, Clinical Effects, Exposure Assessment, Prevention and Control in Indoor Environments and Work. Johanning, E. (ed). Albany, NY: Fungal Research Group Foundation, pp. 292–299.
3. Andersson, M.A., Mikkola, R., Rasimus, S., Hoornstra, D., Salin, P., Rahkila, R., Heikkinen, M., Mattila, S., Peltola, J., Kalso, S., and Salkinoja-Salonen, M. (2010) Boar spermatozoa as a biosensor for detecting toxic substances in indoor dust and aerosols. Toxicology in Vitro, 24(7), 2041–2052. https://doi.org/10.1016/j.tiv.2010.08.011
4. Busse, H.-J., Denner, E.B.M., Buczolits, S., Salkinoja-Salonen, M., Bennasar, A., and Kämpfer, P. (2003) Sphingomonas aurantiaca sp. nov., Sphingomonas aerolata sp. nov. and Sphingomonas faeni sp. nov., air- and dustborne and Antarctic, orange-pigmented, psychrotolerant bacteria, and emended description of the genus Sphingomonas. International Journal of Systematic and Evolutionary Microbiology, 53(5), 1253–1260. https://doi.org/10.1099/ijs.0.02461-0
5. Mikkola, R., Andersson, M.A., Grigoriev, P.A., Teplova, V.V., Saris, N.E., Rainey, F.A., and Salkinoja-Salonen, M.S. (2004) Bacillus amyloliquefaciens strains isolated from moisture-damaged buildings produced surfactin and a substance toxic to mammalian cells. Archives of Microbiology, 181, 314–323. https://doi.org/10.1007/s00203-004-0660-x
